# Analytical and numerical design of a hybrid Fabry–Perot plano-concave microcavity for hexagonal boron nitride

**DOI:** 10.3762/bjnano.13.90

**Published:** 2022-09-27

**Authors:** Felipe Ortiz-Huerta, Karina Garay-Palmett

**Affiliations:** 1 Departamento de Óptica, Centro de Investigación Científica y de Educación Superior de Ensenada, Ensenada, Baja California 22860, México

**Keywords:** Fabry–Perot, hBN, microcavities, plano-concave, polymers

## Abstract

An efficient single-photon emitter (SPE) should emit photons at a high rate into a well-defined spatio-temporal mode along with an accessible numerical aperture (NA) to increase the light extraction efficiency that is required for effective coupling into optical waveguides. Based on a previously developed experimental approach to fabricate hybrid Fabry–Perot microcavities (Ortiz-Huerta et al. *Opt. Express* **2018**, *26*, 33245), we managed to find analytical and finite-difference time-domain (FDTD) values for the, experimentally achievable, geometrical parameters of a hybrid plano-concave microcavity that enhances the spontaneous emission (i.e., Purcell enhancement) of color centers in two-dimensional (2D) hexagonal boron nitride (hBN) while simultaneously limiting the NA of the emitter. Paraxial approximation and a transfer matrix model are used to find the spotsize of the fundamental Gaussian mode and the resonant modes of our microcavity, respectively. A Purcell enhancement of 6 is found for a SPE (i.e., in-plane dipole) hosted by a 2D hBN layer inside the hybrid plano-concave microcavity.

## Introduction

Pure and indistinguishable SPEs are key components needed for their application in upcoming quantum technologies [1] (e.g., quantum computation [2] and quantum networks [3]). Color centers in 2D hBN and diamonds are among the most promising candidates for solid-state single-photon emission at room temperature [4–5]. Nonetheless, in contrast with bulk diamond, the 2D nature of hBN, hosting color centers (i.e., in-plane dipoles), overcomes the necessity for geometrical approaches [6] (i.e., solid immersion lenses [7]) to reduce the angle of emission of the selected SPE.

Challenges still lie ahead for hBN as an ideal SPE [4] and, in order to overcome them, photonic structures such as open-access Fabry–Perot microcavities [8], microdisk resonators [9], and photonic crystals [10–11] have been designed and built around color centers in hBN to increase its spontaneous emission by means of Purcell effect. An alternative and low-cost approach to build photonic structures uses polymers to embed different types of SPEs (e.g., quantum dots [12], molecules [13]) by a process known as two-photon polymerization (2PP) [14] where a photopolymer resist is illuminated with a focused laser at 780 nm and absorbs two photons simultaneously, which triggers a corresponding chemical reaction that solidifies the material to build the desired shape.

**Figure 1:**
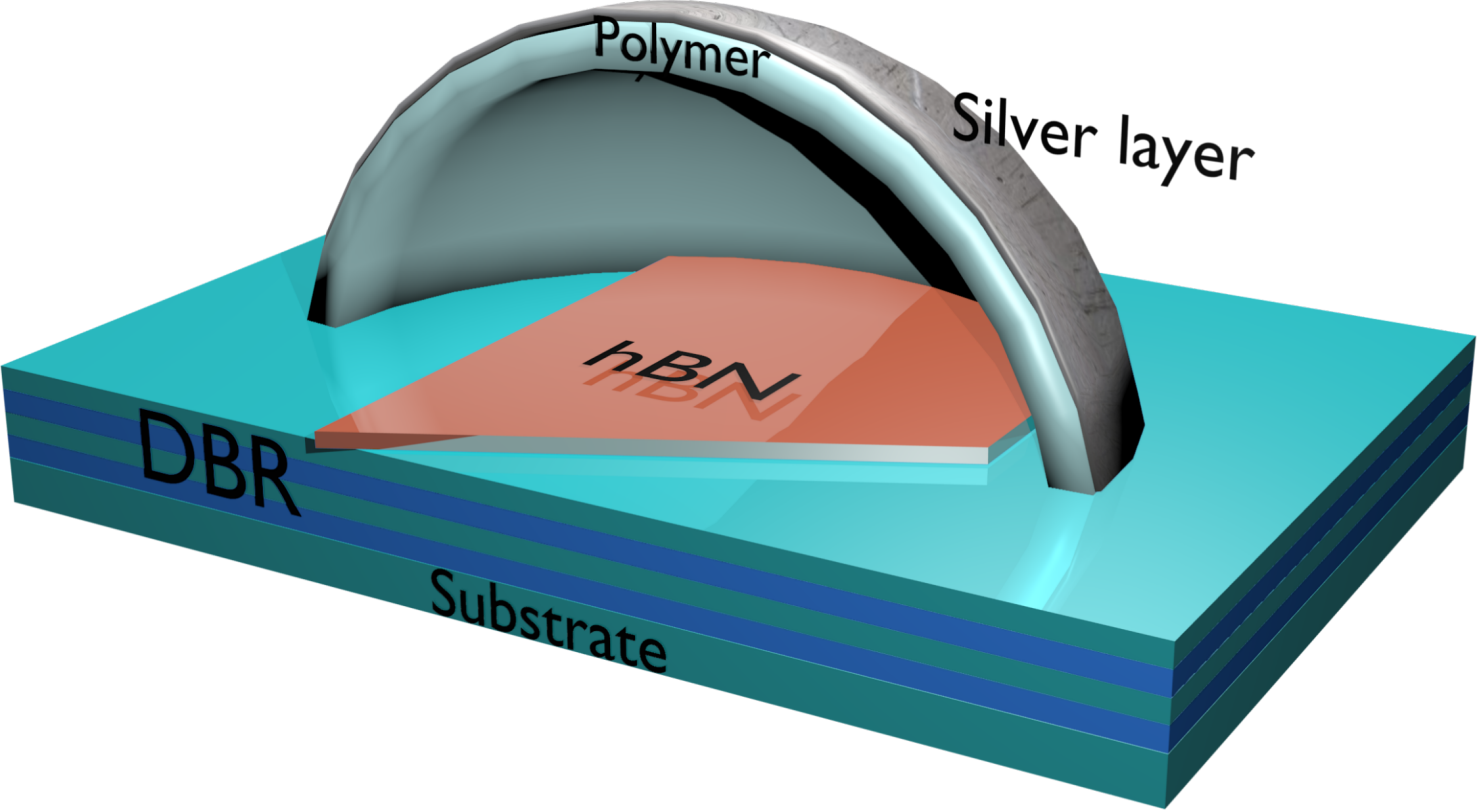
Conceptual design shows cross-section of hybrid plano-concave microcavity with a 2D hBN layer inside on top of a distributed Bragg reflector (DBR).

A natural extension to the development of polymer photonic structures consists of the fabrication of hybrid (i.e., metal-dielectric) resonant structures [15] with the potential to enhance the light–matter interactions of such SPEs. This work will focus on finding an optimal design for a hybrid plano-concave microcavity, containing a multilayer of hBN hosting a SPE (Figure 1), by using analytical methods and FDTD simulations.

Fabrication design steps are first shown for our microcavity, afterwards we found the range of geometrical parameters necessary for our stable resonator, followed by a transfer matrix model used to find the resonant modes of the microcavity, which are then corroborated by FDTD simulations.

## Results and Discussion

### Fabrication design

#### Hybrid plano-concave microcavity

**Figure 2:**
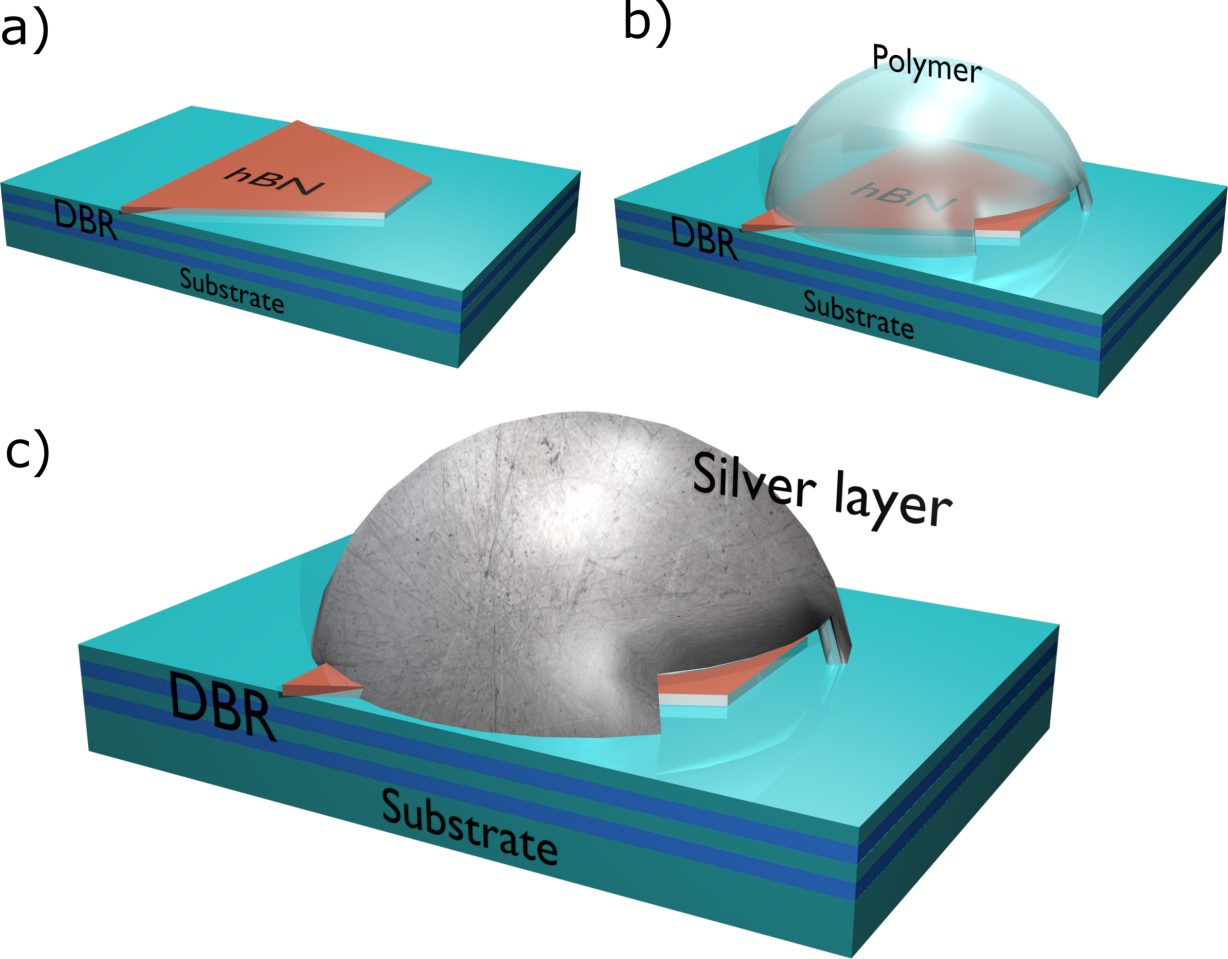
Fabrication steps of hybrid microcavity. (a) hBN layer positioned on top of DBR. (b) Concave polymer shape is fabricated by direct laser writing process. (c) A silver layer is added on top of polymer.

By using a quarter-wavelength DBR with a multilayer 2D material on top (Figure 2a), we designed our system (2D material + DBR stack) to have a maximum reflectivity at the center wavelength of 637 nm. The selected wavelength of our system falls within the typical emission rates of the zero-phonon line (ZPL) of SPEs in hBN (500–800 nm). A quarter-wavelength thickness is conveniently chosen for the hBN where its value falls between experimentally achievable thicknesses of multilayer 2D materials [6].

A 3D concave shape polymer then could be fabricated on top of the 2D material (Figure 2b) by a direct laser writing system (e.g., Photonic Professional, Nanoscribe GmbH) by use of a 2PP process.

Afterwards an 80 nm silver layer could be added, by thermal evaporative deposition, on top of the concave shape polymer to ensure a high reflectivity inside our microcavity. When designing the concave shape polymer a small rectangular aperture at its edge must be taken into account in the fabrication step (Figure 2b,c) to prevent the accumulation of the photopolymer resist, inside the solidified concave polymer, when the sample is developed (SU-8 developer) and cleaned (IPA) to remove any remaining photoresist and developer, respectively, after the 2PP process is finished.

### Analytical design

#### Geometrical parameters of the plano-concave microcavity

**Figure 3:**
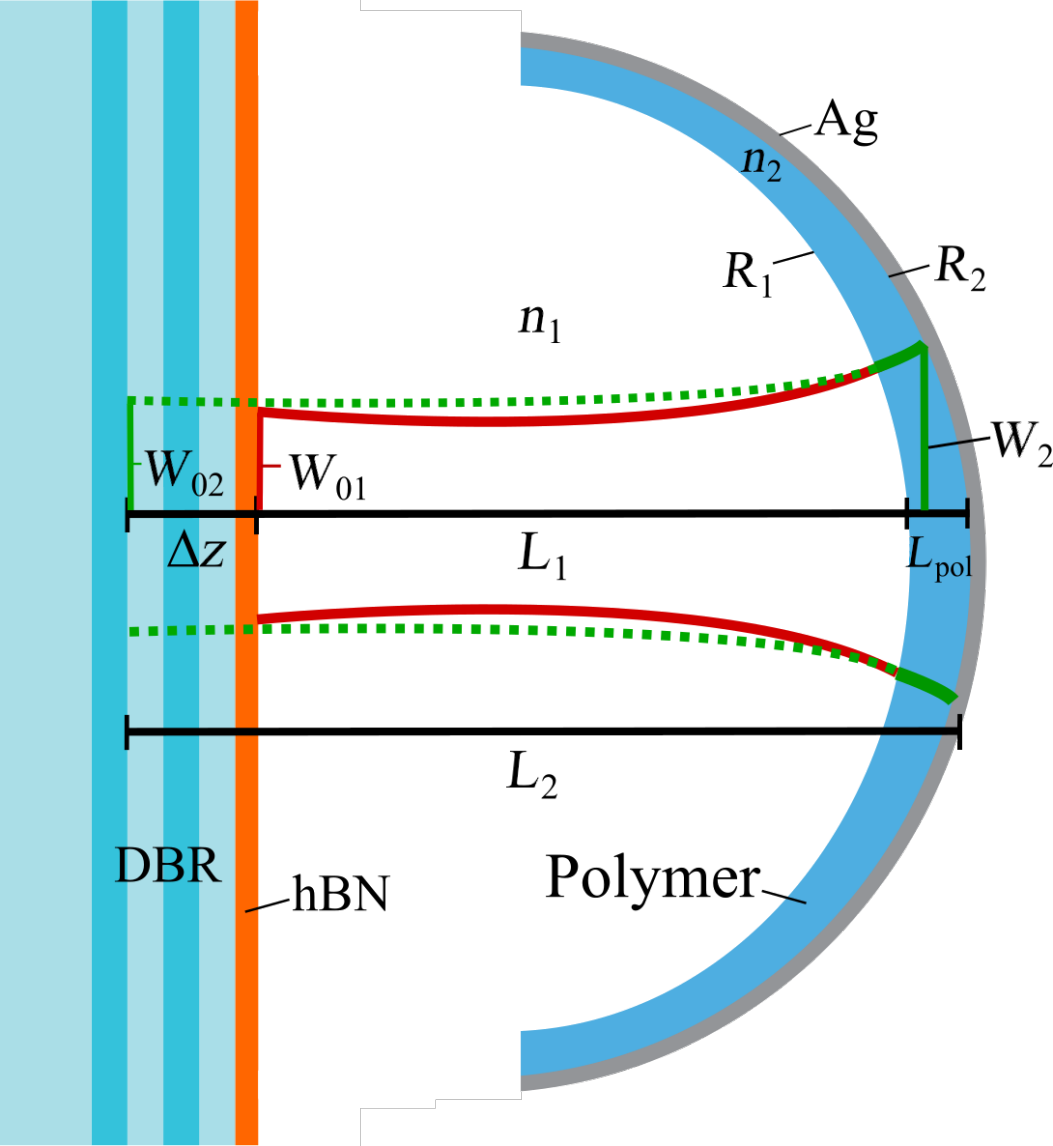
Cross-section of hybrid plano-concave microcavity shows the geometrical parameters and the two Gaussian modes inside.

When a polymer layer is added inside a bare microcavity, as in our case, two fundamental Gaussian beams are formed inside the air gap and polymer layer, respectively (Figure 3) [16].

The spotsize *W*_02_ (Figure 3) of the fundamental Gaussian mode (*TEM*_00_ ) inside the cavity has to be as small as possible, since this means a small modal volume and consequently, a high Purcell factor [17].

**Figure 4:**
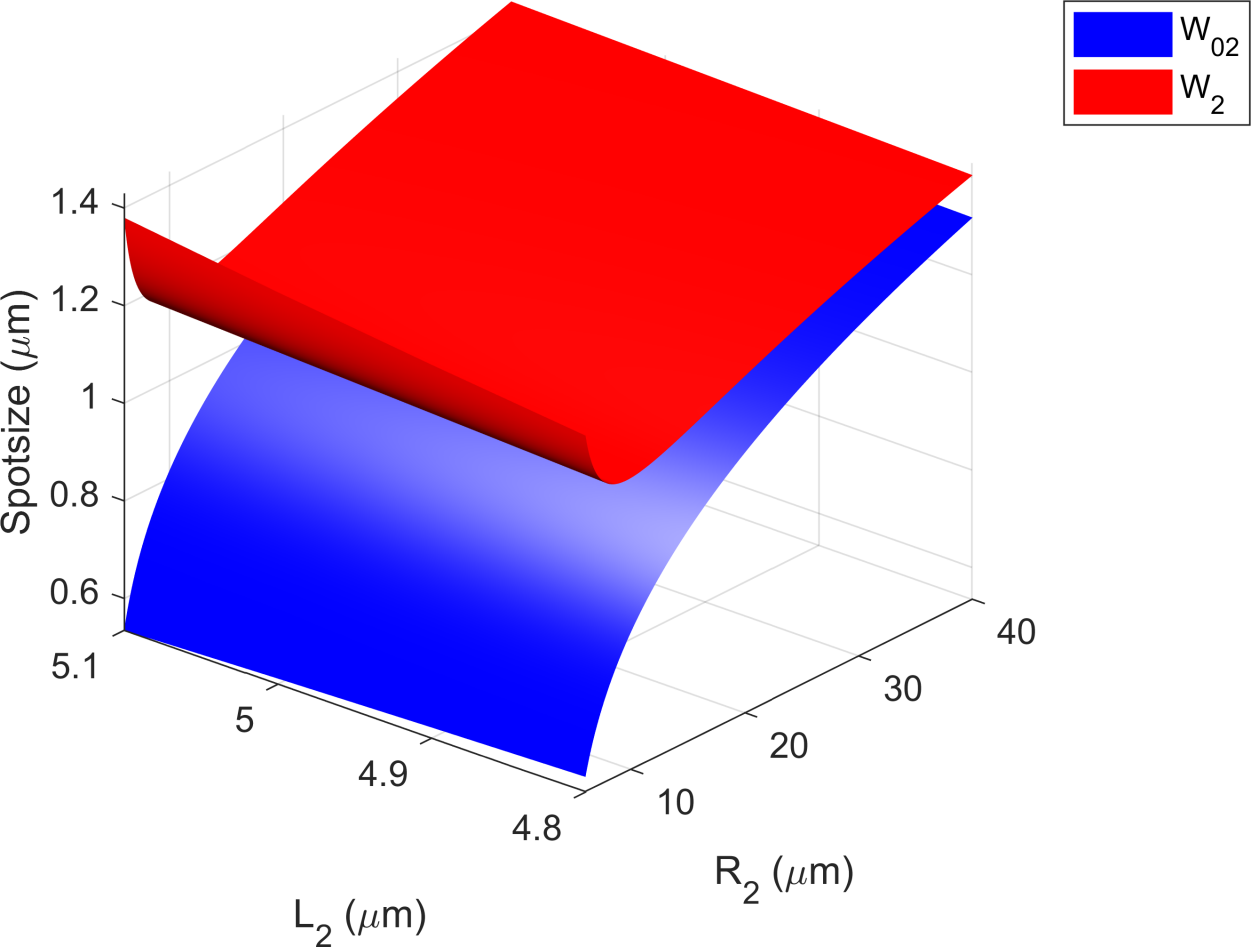
Spotsizes *W*_02_ and *W*_2_ for different values of *R*_2_ and *L*_2_.

By setting an arbitrary range of values for the length of the second Gaussian beam *L*_2_ and radius of curvature *R*_2_ of our plano-concave microcavity, Figure 4 shows the spotsizes *W*_02_ and *W*_2_ corresponding to different pair of values (*R*_2_, *L*_2_) for a hybrid plano-concave cavity. The spotsizes *W*_02_ and *W*_2_ are calculated by [18]:


[1] $$W_{02}^{2}=\frac{L_{2}\lambda_{0}}{\pi n_{2}}\sqrt{\frac{g}{1-g}}$$

and


[2] $$W_{2}^{2}=\frac{L_{2}\lambda_{0}}{\pi n_{2}}\sqrt{\frac{1}{g(1-g)}},$$

respectively, where *g = 1 – L*_2_/*R*_2_ is the stability range for our plano-concave cavity and λ_0_ = 637 nm is the wavelength of the fundamental Gaussian mode, *n*_2_ = 1.52 is the refractive index of the polymer layer. The length of the second Gaussian beam is defined as *L*_2_ = *L*_1_ + *L*_pol_ + ∆*z*, where *L*_1_ is the length of the Gaussian beam in air, *L*_pol_ is the polymer thickness and ∆*z* is calculated by the ABCD law [16]:


[3] $$q_{2}=\frac{Aq_{1}+B}{Cq_{1}+D},$$

where the complex numbers *q*_1,2_ = *z*_1,2_ + *jz*_R,1,2_ are known as the q-parameters for the Gaussian beams, where *z*_2_ = *L*_2_ − *L*_p_, *z*_1_ = *L*_1_ and *z*_R,1,2_ is the Rayleigh length for each beam. For a Gaussian beam passing through a plane dielectric interface, we have *A* = *B* = *C* = 0, and *D* = *n*_2_/*n*_1_, where *n*_1_ = 1 is the refractive index of the air gap, therefore, by substituting in Equation 3, *q*_2_ = (*n*_2_/*n*_1_)*q*_1_. This leads to *z*_2_ = (*n*_2_/*n*_1_)*z*_1_ and *W*_01_ = *W*_02_. Finally, by defining ∆*z* = *z*_2_ − *z*_1_ we get:


[4] $$\Delta z=\left(\frac{n_{2}}{n_{1}}-1\right)L_{1}.$$

As a threshold for *R*_2_ we set *R*_2_ ≥ *L*_2_ in accordance with the stability range where 0 ≤ *g* ≤ 1. Although work has been done to include the lensing effect of a curved “*n*_1_/*n*_2_” interface (see supplementary material of [19]), the planar surface (*R*_1_ = ∞) approximation values (Table 1) fall within the desired range with our FDTD simulations.

**Figure 5:**
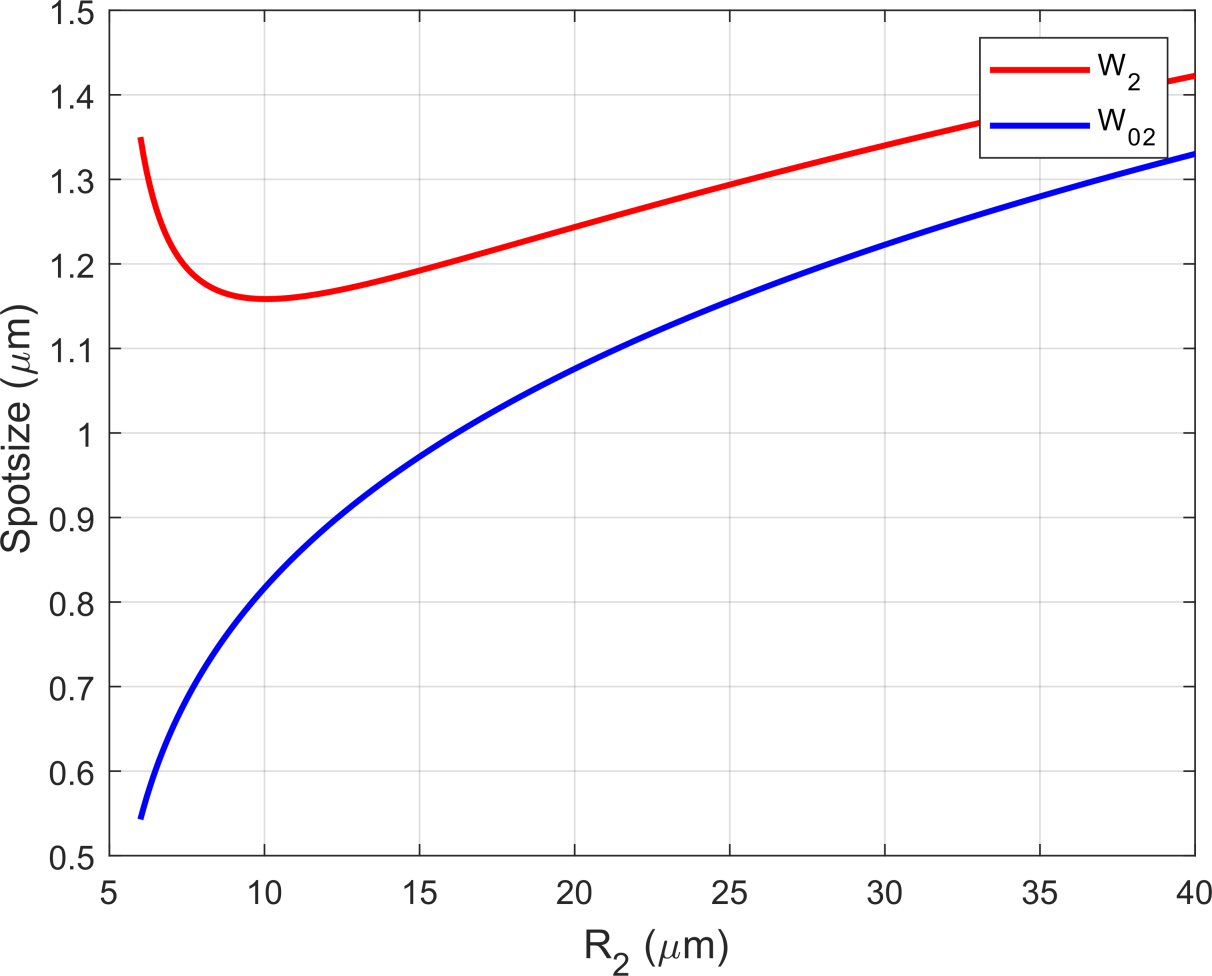
Transverse cut of Figure 4 through length *L*_2_ = 5.03 μm to show dependence of *R*_2_ with spotsizes. As the values of *R*_2_ diminishes, while maintaining a constant *L*_2_, the functions for *W*_02_ (blue) and *W*_2_ (red) start to diverge, arriving at the limit of the paraxial approximation (stability regime).

We take a transversal cut through a fixed value of *L*_2_ (Figure 5) and observe the dependence of *W*_02_ and *W*_2_ to the radius of curvature (*R*_2_) of a plano-concave cavity. To achieve a high Purcell factor, and a small NA, *R*_2_ must be as small as possible (small *W*_02_), while maintaining the lower boundary condition (*R*_2_* ≥ L*_2_)*,* therefore the optimal values of *R*_2_, for any arbitrary *L*_2_, will reside near the vicinity of the minima of the *W*_2_ function (Figure 5), setting the boundary values for *R*_2_, for any given *L*_2_, at *R*_2_ ≈ 2*L*_2_.

Selecting the *R*_2_ parameter closer to the divergence of the *W*_2_ function (*R*_2_ = *L*_2_) could result in unstable resonators that will not hold a stable Gaussian mode inside. Theoretical work has been done with *R*_2_ ≈ *L*_2_ [20], where a non-paraxial analysis is performed, although diffraction losses have to be considered for an accurate description of the experimental limits of stability [21]. In the unstable regime (*R*_2_ < *L*_2_) extensive work has also been done [22–23].

#### Electric field distribution and resonant modes of the plano-concave microcavity

**Figure 6:**
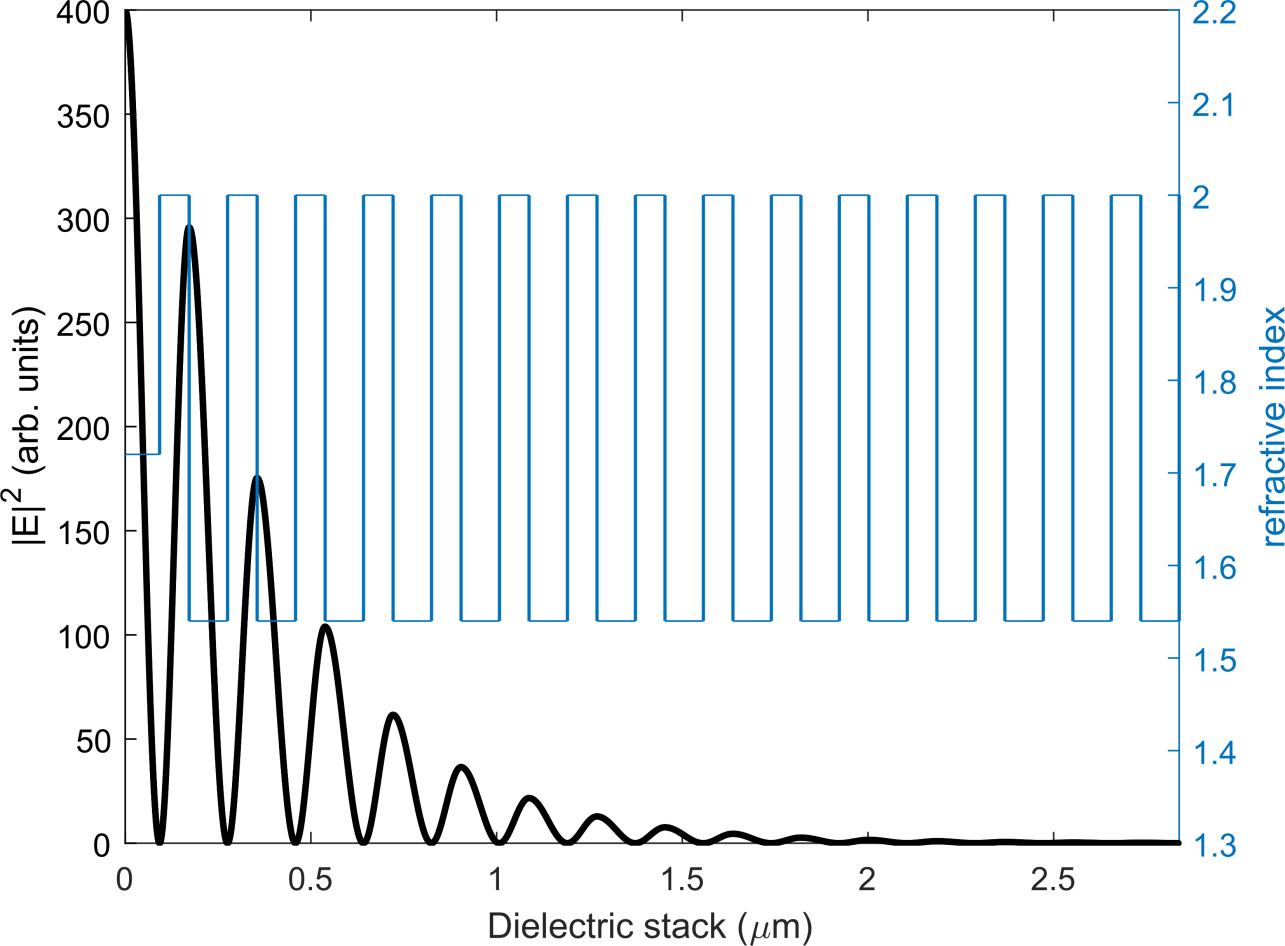
Electric field distribution of a hBN + DBR system on a *L*(*HL*)^15^ configuration. Maximum electric field intensity is found at the surface of the hBN layer. Vertical lines (blue) represent the boundaries between each dielectric layer.

A λ_0_/4*n* thickness layer of hBN (*n* = 1.72) was positioned on top of a 15-pair layer DBR with tantalum oxide (Ta_2_O_5_) and silicon oxide (SiO_2_) as the high- and low-index layers, respectively, on a (*HL*)^15^ configuration to ensure an electric field antinode at the surface of the hBN layer, making the hBN + DBR system a *L*(*HL*)^15^ dielectric stack. A transfer matrix model [24] was used to calculate the electric field distribution inside the hBN + DBR system (Figure 6).

The full transfer matrix *S* of our microcavity is defined as:


[5] $$S=L_{\text{Ag}}I_{1}L_{\text{pol}}I_{2}L_{\text{air}}I_{3}L_{\text{hBN}}I_{4}L_{\text{DBR}}I_{5},$$

where *L* and *I* represent the transfer and interface matrix, respectively, of the silver (Ag), polymer (pol), air, hBN and DBR layer. The transfer matrices *L*_pol_ and *L*_air_ are defined as [25]:


[6] $$L_{\text{pol}}=\left[\begin{array}{cc} \exp\left(-\frac{i2\pi n_{2}}{\lambda_{0}}+iG_{2}\right) & 0\\ 0 & \exp\left(-\frac{i2\pi n_{2}}{\lambda_{0}}+iG_{2}\right) \end{array}\right],$$

[7] $$L_{\text{air}}=\left[\begin{array}{cc} \exp\left(-\frac{i2\pi n_{1}}{\lambda_{0}}+iG_{1}\right) & 0\\ 0 & \exp\left(-\frac{i2\pi n_{1}}{\lambda_{0}}+iG_{1}\right) \end{array}\right],$$

where *G*_1,2_ = arctan(*L*_1,2_λ_0_/*n*_1,2_π*W*_01,02_) is the Guoy phase shift in the air (*n*_1_ = 1) and polymer layer, respectively, where *W*_01_ = *W*_02_.

**Figure 7:**
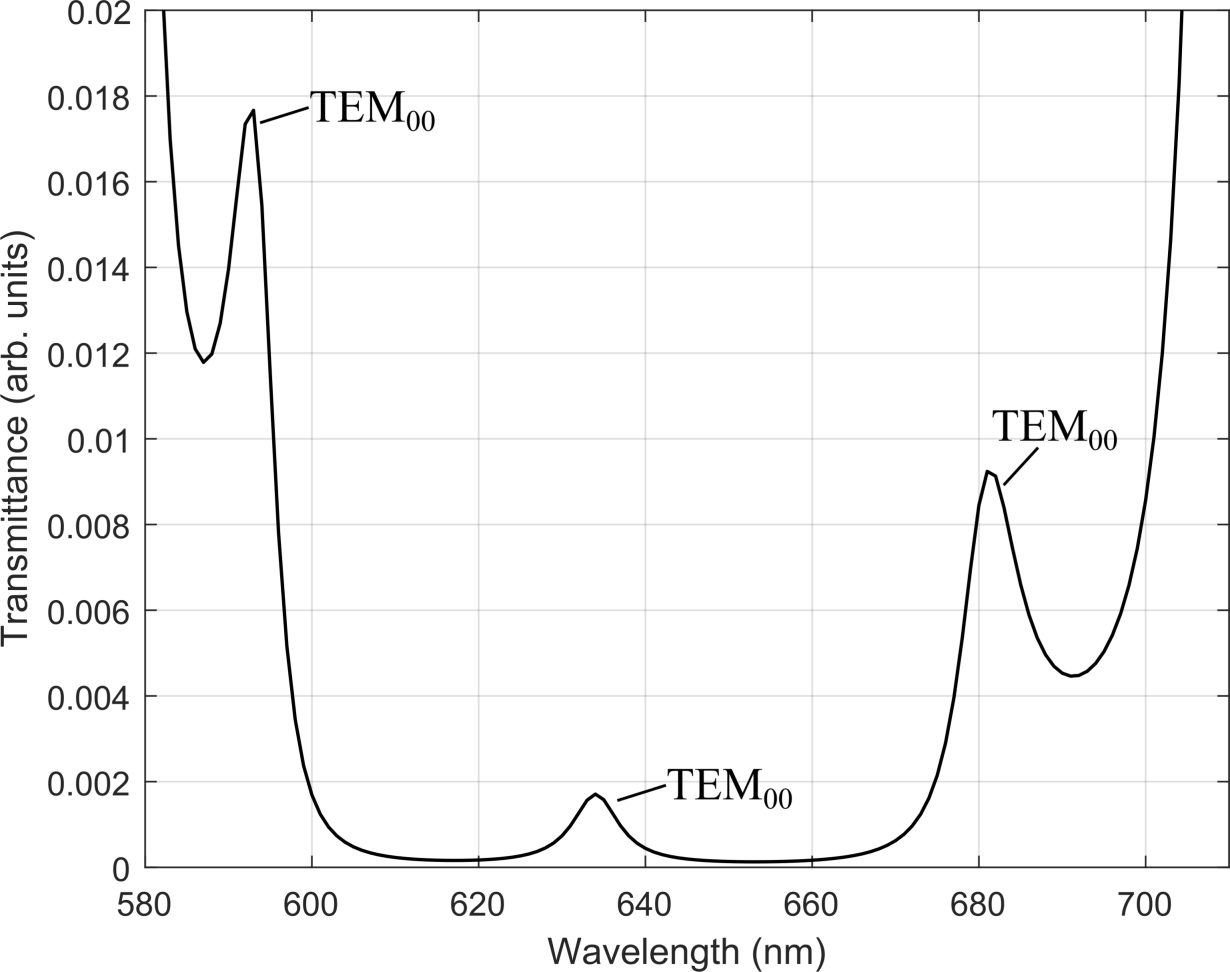
Transmittance of plano-concave cavity shows the fundamental TEM modes at 595 nm, 636 nm and 684 nm.

The transmittance of the microcavity is calculated, from the matrix elements of *S*, to find its fundamental TEM resonant modes (Figure 7). We found the desired TEM modes at *R*_2_ = 8.1 μm and *L*_2_ = *L*_1_ + *L*_pol_ + ∆*z* = 5.03 μm, where *L*_1_ = 3.09 μm, *L*_pol_ = 0.4 μm and ∆*z* = 1.54 μm, which gives a physical cavity length of *L = L*_2_ − Δz = 3.49 μm. These values fall within the stability range *R*_2_ ≈ 2*L*_2_.

### Numerical design

#### Resonant modes of hybrid plano-concave microcavity

**Figure 8:**
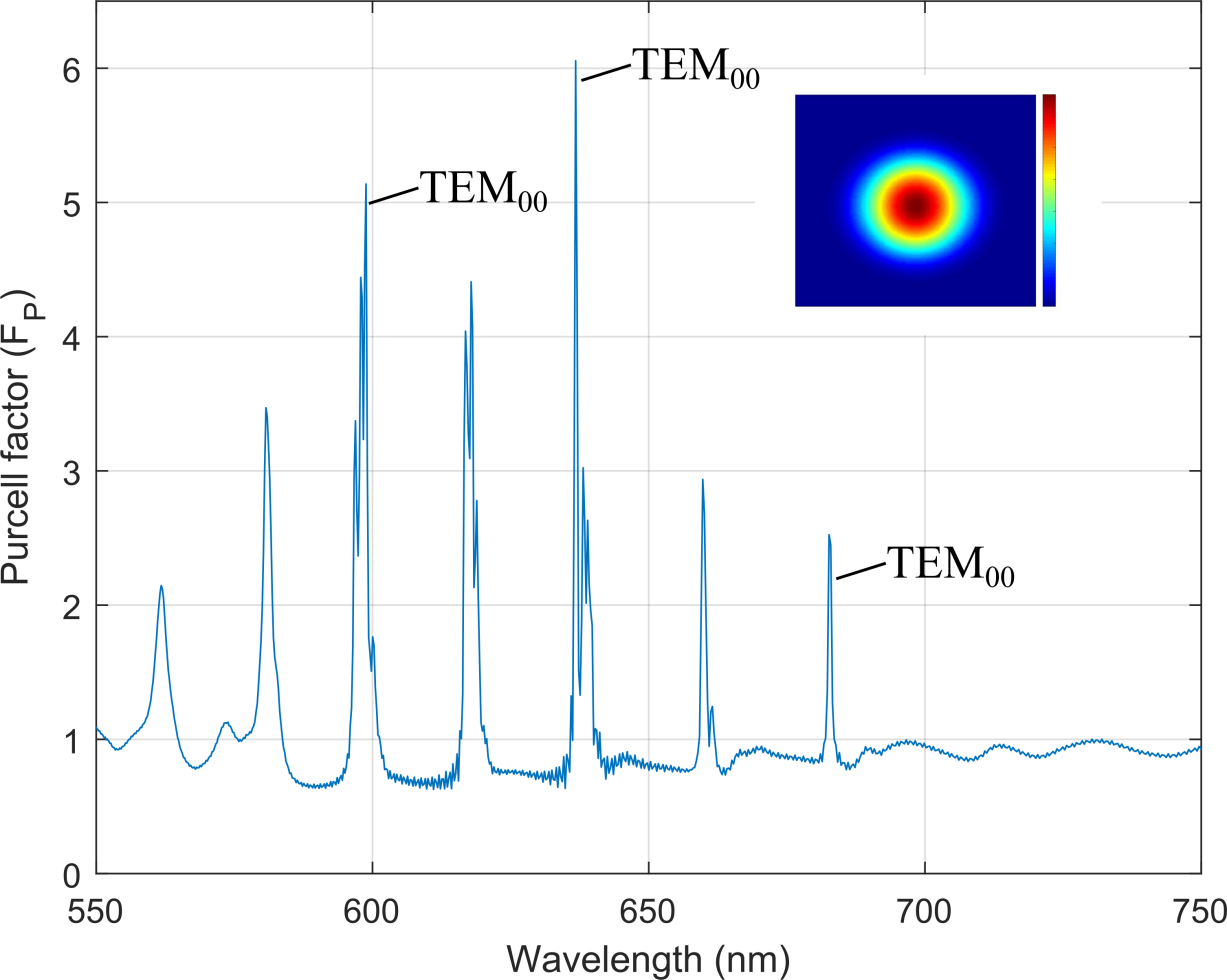
Purcell factor of plano-concave microcavity. Fundamental TEM Gaussian modes are found at 595 nm, 636 nm and 684 nm. Inset shows transverse section of fundamental Gaussian mode at 637 nm.

For the FDTD simulations, we used the Ansys Lumerical FDTD software. The polymer, and DBR stack were treated as lossless and non-dispersive materials [15]. A transmittance *T* = 8% at 637 nm is measured for our cavity, with an in-plane dipole inside, for a silver layer thickness of 80 nm. Identical values for the geometrical parameters previously mentioned (*R*_2_, *L*_2_, *L*_1_), except for *R*_1_ = 7.7 μm, were taken for the FDTD simulations, where an in-plane dipole emitter sits at the surface of the hBN layer to ensure a higher Purcell factor since the dipole interacts with an electric field antinode [26]. The Purcell factor was calculated by using the classical definition [27]:


[8] $$F_{\text{p}}=\frac{P_{\text{cav}}}{P_{\text{free}}},$$

where *P*_cav_ and *P*_free_ is the power dissipated for the dipole inside the microcavity and in free space, respectively. A Purcell factor of *F*_P_ ≈ 6 was achieved for the TEM mode at the DBR center wavelength. A Q-factor of *Q* = 731.4 ± 102.7 was also calculated in our simulations where the resonant modes of the microcavity (Figure 8) are shown in good agreement (Table 1) with the resultant modes from the analytical model (Figure 7).

**Table 1:** Geometrical parameters and fundamental TEM mode values of the designed hybrid plano-concave microcavity.

Parameter	Analytical (μm)	FDTD (μm)

*R*_2_	8.1	8.1
physical cavity length, *L*	3.49	3.49
*L*1	3.09	3.09
*L*2	5.03	5.03
hBN thickness	λ_0_/4*n*	λ_0_/4*n*
polymer thickness	0.4	0.4
1st *TEM*_00_	0.595	0.616
2nd *TEM*_00_	0.636	0.637
3rd *TEM*_00_	0.684	0.684
*R*_1_	∞	7.7

## Conclusion

We have presented the fabrication design steps for a new type of hybrid plano-concave microcavity and found its fundamental resonant modes by using an expanded transfer matrix model to account for the curvature in dielectrics and, by using FDTD simulations, we were able to show the effectiveness of the analytical model and found a Purcell enhancement of 6 for a pre-selected SPE.

The geometrical parameters of our microcavity are all experimentally achievable with the two-photon absorption fabrication process [13,15] and our modeled cavity could easily be extended to contain and enhance spontaneous emission of arbitrary solid-state SPEs [28]. Although novel approaches have been realized to diminish vibrations for open-access Fabry–Perot microcavities inside a cryostat system [29], in our design, the plano-concave microcavity is integrated directly to the substrate containing the SPE and, therefore, there are no moving parts that could potentially diminish the Purcell factor of a pre-selected SPE due to vibrations in cavity length [30], although detuning of the selected mode, due to thermally-induced contraction of the polymer by cooling [12], must be taken into account if the desired SPE and the cavity are to be analyzed inside a cryostat system.

The methodology of design of the hybrid Fabry-Perot microcavity is also suited for quantum cryptography applications, provided the emitter’s wavelength is within the telecom range [6], and potential chemical sensing applications [31], since our microcavity is also an open-access cavity.
